# Survival in Nuclear Waste, Extreme Resistance, and Potential Applications Gleaned from the Genome Sequence of *Kineococcus radiotolerans* SRS30216

**DOI:** 10.1371/journal.pone.0003878

**Published:** 2008-12-05

**Authors:** Christopher E. Bagwell, Swapna Bhat, Gary M. Hawkins, Bryan W. Smith, Tapan Biswas, Timothy R. Hoover, Elizabeth Saunders, Cliff S. Han, Oleg V. Tsodikov, Lawrence J. Shimkets

**Affiliations:** 1 Savannah River National Laboratory, Environmental Sciences and Biotechnology, Aiken, South Carolina, United States of America; 2 Department of Medicinal Chemistry, College of Pharmacy, University of Michigan, Ann Arbor, Michigan, United States of America; 3 Department of Microbiology, University of Georgia, Athens, Georgia, United States of America; 4 DOE Joint Genome Institute, Bioscience Division, Los Alamos National Laboratory, Los Alamos, New Mexico, United States of America; Centre for DNA Fingerprinting and Diagnostics, India

## Abstract

*Kineococcus radiotolerans* SRS30216 was isolated from a high-level radioactive environment at the Savannah River Site (SRS) and exhibits γ-radiation resistance approaching that of *Deinococcus radiodurans*. The genome was sequenced by the U.S. Department of Energy's Joint Genome Institute which suggested the existence of three replicons, a 4.76 Mb linear chromosome, a 0.18 Mb linear plasmid, and a 12.92 Kb circular plasmid. Southern hybridization confirmed that the chromosome is linear. The *K. radiotolerans* genome sequence was examined to learn about the physiology of the organism with regard to ionizing radiation resistance, the potential for bioremediation of nuclear waste, and the dimorphic life cycle. *K. radiotolerans* may have a unique genetic toolbox for radiation protection as it lacks many of the genes known to confer radiation resistance in *D. radiodurans*. Additionally, genes involved in the detoxification of reactive oxygen species and the excision repair pathway are overrepresented. *K. radiotolerans* appears to lack degradation pathways for pervasive soil and groundwater pollutants. However, it can respire on two organic acids found in SRS high-level nuclear waste, formate and oxalate, which promote the survival of cells during prolonged periods of starvation. The dimorphic life cycle involves the production of motile zoospores. The flagellar biosynthesis genes are located on a motility island, though its regulation could not be fully discerned. These results highlight the remarkable ability of *K radiotolerans* to withstand environmental extremes and suggest that *in situ* bioremediation of organic complexants from high level radioactive waste may be feasible.

## Introduction

High-level radioactive waste (HLW) is an anthropogenic disturbance to which few organisms are resistant. During the Cold War, Pu^239^ production for national defense began by irradiating uranium or other target elements in a nuclear reactor. At Hanford, WA and the Savannah River Site (SRS), SC, irradiated fuel and targets were reprocessed to reclaim approximately 99% of the U^235^ and Pu^239^ isotopes. All remaining radionuclides, fission products, fuel components, and nonradioactive chemicals used during reclamation make up the HLW, which currently resides at over 100 different sites across the contiguous U.S. and exceeds 1 billion curies. The majority of this lasting Cold War legacy is located at Hanford (roughly 65 million gallons) and SRS (roughly 35 million gallons).

The SRS waste contains Fe, Al, Si, Ca, F, K, alkali cations, organic solvents, radionuclides, and other fission products. Organic constituents include complexants used during separations, radiolysis products from degradation of complexants and solvents, and waste tank decontamination reagents. One of the preferred decontamination reagents is oxalic acid, which can create problems for storage and final disposal due to its solubility properties as a sodium salt. Additional reagents in use at SRS and Hanford include glycolic acid, citric acid, and formic acid. Removal of organic constituents directly in HLW tanks could greatly improve processing efficiency of HLW.

In 2001, the Committee on Long-Term Research Needs for Radioactive High-Level Waste at Department of Energy Sites recommended the investigation of radioactive waste to identify promising new radiation resistant microorganisms that might be used to degrade some of the organic constituents in the HLW. Radiation dosage is measured in gray (Gy); 1 Gy causes the first signs of radiation sickness in humans. Half of all people exposed to 4.5 Gy die, and doses of 8 Gy or more are invariably fatal to humans. Bacteria are the most extreme examples of radiation resistant organisms. The paradigm is *Deinococcus radiodurans*, which was isolated from canned meat that received 4,000 Gy of ionizing-radiation [1], but many other radiation resistant bacteria have also been identified.

*Kineococcus radiotolerans* SRS30216 was isolated from HLW within a shielded cell work area at the SRS [2]. *K. radiotolerans* is an orange-pigmented, aerobic bacterium belonging to the Actinobacteria phylum that is capable of withstanding relatively high concentrations of metals and alkali cations, as well as exposure to extreme doses of ionizing radiation. *K. radiotolerans* exhibits radiation resistance approaching that of *D. radiodurans* [2]. *D. radiodurans* and *K. radiotolerans* belong to different phyla and it remains unknown whether both organisms attain radiation resistance through a common set of gene products. Because *K. radiotolerans* survived a HLW environment, it is expected to possess potent cellular defense and repair mechanisms for radiation exposure, osmotic stress and chemical toxicity. The occurrence of all these features in a naturally occurring bacterium may have direct applications for the bioremediation of nuclear waste.

In this work the genome sequence of *K. radiotolerans* SRS30216 was examined in three different contexts. First, the presence of genes known to confer radiation resistance in *D. radiodurans* was examined in the *K. radiotolerans* genome. Second, the capacity for bioremediation was assessed by comparative genomics as well as growth and respiration studies. Finally, the dimorphic life cycle of the organism, in particular the production of motile zoospores, was examined by identifying genes involved in flagellar motility and chemotaxis.

## Results and Discussion

### Phylogeny

*K. radiotolerans* belongs to the suborder Frankineae in the order Actinomycetales and the phylum Actinobacteria. Complete genome sequences are available for only two other members of the Frankineae suborder, *Frankia alni* and *Acidothermus cellulolyticus*. *F. alni* is distinguished by its ability to fix nitrogen in symbiosis with alder (*Alnus* spp.) and myrtle (*Myrica* spp.), two pioneer plants in temperate regions. The *K. radiotolerans* genome shows no potential for nitrogen fixation. *A. cellulolyticus* is a thermotolerant organism isolated from the hot spring in Yellowstone National Park that degrades cellulose. While the *K. radiotolerans* genome may encode proteins with the potential for degrading complex carbohydrates like cellulose (Krad4622, 3823), cellobiose (Krad0408, 2526, 2530, 2531, 2539, 3436, 3480, 3961), glycogen, and starch (Krad1294, 1298), growth on these substrates has not been demonstrated experimentally.

More distant relatives in the same phylum include *Streptomyces* and *Mycobacterium*, whose physiology has been extensively examined, and which serve as reference points for comparative genomics.

### Genome size and organization

The *K. radiotolerans* SRS30216 genome was sequenced by the US DOE Joint Genome Institute with the discovery of three replicons. The bulk of the DNA is contained on a 4,761,183 bp linear chromosome (CP000750). In addition there is a 182,572 bp linear plasmid (pKRAD01; CP000751) and a 12,917 bp circular plasmid (pKRAD02; CP000752). The three contigs were derived from at least 20 reads and average 74.2 mol % G+C. The genome is predicted to contain 4,715 genes.

**Figure 1:**
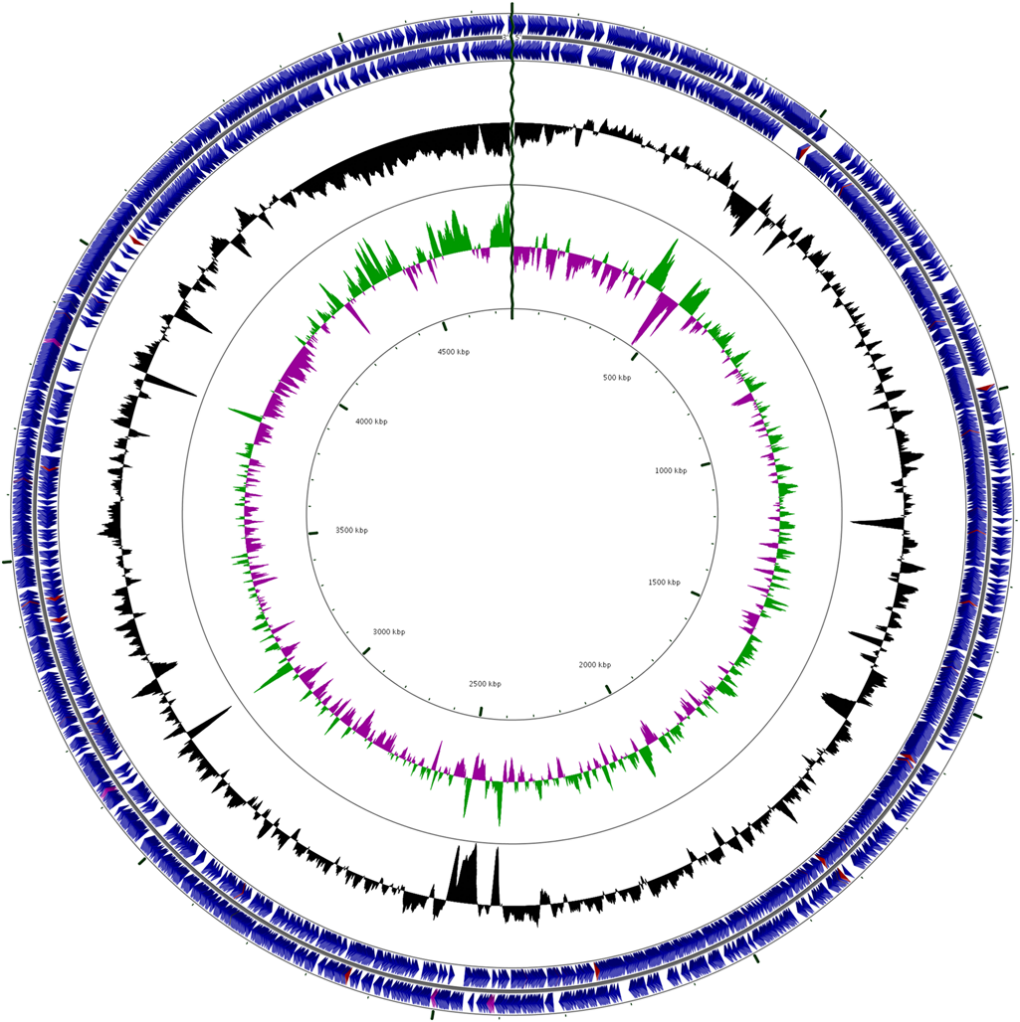
Gene distribution of the 4.76 Mb *Kineococcus radiotolerans* SRS30216 linear chromosome depicted in circular form. The break is indicated by a wavy line at 12 o'clock. From the outer to the inner concentric circle: circles 1 and 2, predicted protein coding sequences (CDS) indicated by blue arrows on the forward (outer wheel) and reverse (inner wheel) strands. Red indicates tRNA genes and purple indicates rRNA genes; circle 3, GC content showing deviation from average (74.2%); circle 4, GC skew (+ is green and − is purple); circle 5, genomic position in kb beginning with Krad2223 and proceeding clockwise. The chromosome map was generated using CGview [56].

Linear chromosomes are rare among prokaryotes. Aside from one atypical *Agrobacterium* isolate, the two principle examples are *Streptomyces* species [3] and *Borrelia burgdorferi* [4]. In both *Streptomyces* and *Borrelia* bidirectional replication occurs from a single origin of replication (*oriC*) located near the middle of the replicon. A plot of the GC skew = (C−G)/(C+G) along the chromosome sometimes inverts at the replication origin [5]. The shift in GC skew is thought to be due to accumulated mutational biases during leading vs lagging strand synthesis. There is no obvious GC skew at the center of the chromosome (Figure 1, compare green vs magenta peaks in inner circle). There is however a remarkable GC skew at the telomers but it seems unlikely that replication proceeds from the telomers toward the center based on the *Borrelia* and *Streptomyces* systems. A second method used to locate *oriC* is the presence of the *dnaA* gene and DnaA binding sites. *dnaA* (Krad0001) is near the center of the chromosome, as with *Streptomyces* and *Borrelia.* Putative, imperfect blocks of DnaA binding sites, observed using DoriC [6], are widely scattered in the center of the chromosome with the closest being about 94 Kb from the *dnaA* gene.

**Figure 2:**
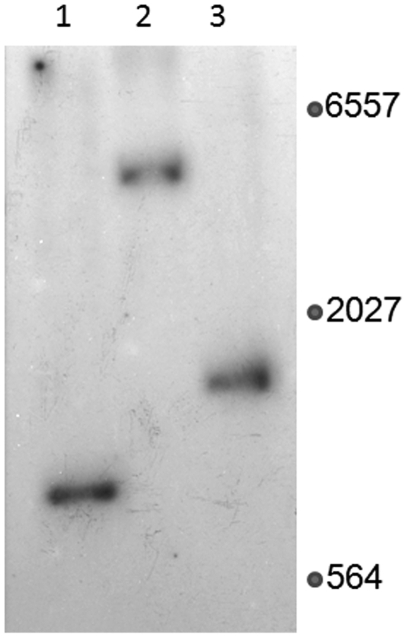
Southern hybridization reveals a linear chromosome. PCR was used to generate probes for the first end in the presumptive linear chromosome homologous with a portion of the first gene Krad2223. The genome was digested with one of three restriction enzymes predicted from the DNA sequence to generate a hybridization product with each probe. Lane 1, NcoI; Lane 2, BamHI; Lane 3, BglII. Sizes of molecular markers in base pairs are given in the right hand column. The predicted sizes of the homologous restriction fragments from the DNA sequence are NcoI, 865 bp, BamHI, 3692 bp, and BglII, 1619 bp with the Krad2223 probe. The predicted sizes of the restriction fragments from the other end of the chromosome are NcoI, 775 bp, BamHI, 1363 bp, and BglII, 4018 bp. The observed sizes were calculated using bacteriophage lambda DNA standards digested with HindIII. In all cases the observed sizes were approximately equal to the sizes predicted from a linear chromosome rather than the sum of the sizes from both ends suggesting a linear topology.

The topology of the chromosome was investigated by Southern hybridization using an end-specific probe homologous with Krad2223. Three restriction enzymes were predicted to generate single fragments with this probe. If the chromosome is linear then products with sizes predicted from the DNA sequence should be visible after hybridization. If the chromosome is circular then the hybridization products with the probe should be the sum of the sizes of the predicted restriction fragments from each end plus any missing sequence. Restriction fragments homologous with the Krad2223 probe were similar in size to those predicted by the DNA sequence (Fig. 2). These hybridization results confirm the linear topology predicted by the inability of JGI to close the DNA sequence.

The ends of linear DNA replicons have special features that preserve their integrity. *B. burgdorferi* linear replicons contain covalently closed hairpin ends [4]. ResT, telomere resolvase, hydrolyzes a phosphodiester bond on each DNA strand then joins the opposite strands to form a covalently closed telomere. The process is reversed during chromosome replication and ResT is essential for *B. burgdorferi* growth. A ResT homolog is not encoded by the *K. radiotolerans* genome. *Streptomyces coelicolor* replicon ends are composed of single-stranded sequences that can anneal to form a noncovalent circular molecule [3]. *Streptomyces* telomeres bind a family of conserved terminal binding proteins that have no orthologs in the *K. radiotolerans* genome. Thus, unique mechanisms must protect the *K. radiotolerans* telomers.

### Ionizing radiation resistance

Gamma radiation is one of the most energetic forms of electromagnetic radiation. Gamma rays penetrate tissues and cells, causing direct damage to DNA (namely double strand breaks), proteins, and membranes. Gamma radiation also induces indirect cellular damage through the ionization of water with formation of free radical species, primarily •OH. Oxygen free radicals are extremely reactive, compounding cellular and DNA damage. DNA damage blocks transcription and replication, and if not correctly repaired, could introduce detrimental mutations or cause cell death. Relatively few DNA double strand breaks (DSB) are lethal for most bacteria. *Escherichia coli* cells succumb to around 10 DSB and *Shewanella oneidensis* cells die after 1 DSB (based on calculations of 0.0114 DSBs/Gy/Genome; Daly et al., 2004).

**Figure 3:**
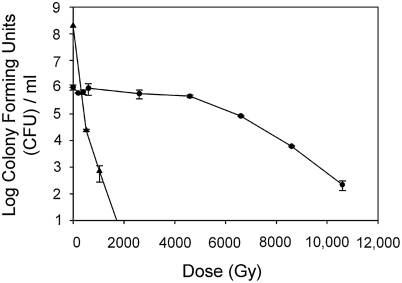
Resistance of *K. radiotolerans* to acute γ-radiation exposure. *E. coli* was used as a reference strain. Prior to irradiation, both strains were grown to exponential phase in TGY and LB, respectively. Colony forming units determinations were conducted in triplicate.

**Figure 4:**
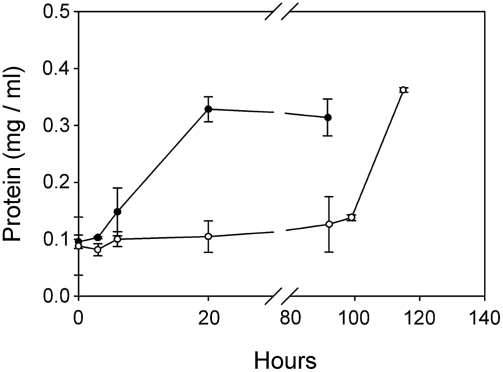
Post-irradiation recovery and growth of *K. radiotolerans*. The irradiated cultures were exposed to 20 kGy γ-radiation (open circles), and control cultures were incubated under laboratory conditions (closed circles).

Radioresistance has been partially characterized for *K. radiotolerans* (Figure 3). Acutely irradiated, exponentially grown cultures have a broad shoulder of death, which contrasts with the exponential death of *E. coli*. This shoulder is due in part to efficient repair systems and in part to the multicellular nature of the organism. In rich medium *K. radiotolerans* grows in cubical packets that form by alternating cell division planes (Phillips et al. 2002). Thus, the colony forming unit method used to estimate survivorship likely overestimates culture viability. Nevertheless, these results may portray a more ecologically relevant context as cell clustering is common for certain species or developmental stages of Actinobacteria (eg., *Frankia*, *Geodermatophilus*, *Actinoplanes, Micrococcus*) or other extremophiles (*Kocuria, Deinococcus, Chroococcidiopsis*). Remarkably though, *Kineococcus* can withstand the damaging effects of 20 kGy of γ-radiation (theoretically generating more than 200 DSB/genome; Daly et al., 2004) and cell division resumes within 4 days (Figure 4).

Several other Actinobacteria species exhibit extraordinary radiation resistance including *Rubrobacter radiotolerans*, *Rubrobacter xylanophilus*, and *Kocuria rosea* [7]. While more ionizing radiation resistant bacterial species are found in the Actinobacteria than any other phylum, radiation resistance is not a widespread trait in this phylum and may have multiple evolutionary origins. The mechanisms that render this trait remain poorly understood. Radiation resistant bacteria suffer severe damage from γ-radiation [8], which implies that molecular repair processes function with high efficiency.

Analysis of genes common to four ionizing radiation resistant bacteria with fully sequenced genomes indicated that DNA repair must have played a major role in evolutionary adaptation to ionizing radiation [9]. *D. radiodurans* has served as the paradigm for radiation resistant organisms and both genetic and biochemical approaches are converging to reveal a complex network of repair and protection processes [7], [10]. Many *D. radiodurans* genes required for ionizing radiation resistance have been identified. In table 1 they are ordered according to the γ-radiation sensitivity of a *D. radiodurans* strain lacking that gene based on published kill curves. While the roles of only a few of these genes are known with certainty, *K. radiotolerans* apparently employs a different genetic toolbox from that of *D. radiodurans*. Absent from *K. radiotolerans* are homologs of the *D. radiodurans pprA*, *ddrA, ddrB, ddrC, ddrD* genes [11].

**Table 1:** Genes conferring ionizing radiation resistance in *Deinococcus radiodurans* and their homologs in *Kineococcus radiotolerans*.

*D. radiodurans* gene	Function	D_10_ (kGy)^1^	Reference	Krad locus tag
*recA*	Homologous recombination	0.1	[11]	1492
*polA*	DNA polymerase	1.0	[24]	2951
*pprA*	Stimulates DNA ligase	2.0	[57]	none
*recQ*	DNA helicase	6.0	[58]	0829
*recD*	Helicase/exonuclease	6.0	[59]	0992
*ddrB*	Unknown	8.0	[11]	none
*crtB*	Phytoene synthase	9.0	[13]	3229
*crtI*	Phytoene desaturase	9.0	[13]	3228
*ddrA*	ssDNA binding protein	12.0	[11]	none
*ddrC*	Unknown	>14.0	[11]	none
*ddrD*	Unknown	>14.0	[11]	none
*sbcC sbcD*	ss endonucleases 3′-5′ ds exonuclease	15.0	[60]	2553 2554
*polX*	ss endonucleases 3′-5′ ds exonuclease	15.0	[61]	4036

1 Dose of γ radiation required for a 90% reduction in cell viability estimated from data supplied in the relevant reference. For comparison, the D_10_ for wild *D. radiotolerans* strains ranges from 10–20 kGy depending on the strain and the assay conditions.

Mutations in *recA* render *D. radiodurans* as sensitive to ionizing radiation as *E. coli* illustrating the importance of recombinational repair in repairing DSB (table 1). *D. radiodurans* cells exposed to 10 kGy γ-radiation accumulate about 100 DSB per genome that are repaired over the course of several hours. *D. radiodurans* DSB are repaired by homologous recombination in the extended synthesis-dependent strand annealing (ESDSA) process [12]. ESDSA repair is carried out by RecA and PolA, enzymes found in the *K. radiotolerans* genome (table 2). Based on their genomic analysis, *recA* and *polA* exhibit signs of coevolution in ionizing radiation-resistant bacteria [9].

**Table 2:** Genes coding for replication, repair and recombination functions in *E. coli, D. radiodurans, M. tuberculosis* and *K. radiotolerans.*

Protein name	Protein description and comments^B^	*Escherichia coli*	*Deinococcus radiodurans* R1 (locus tag)	*Mycobacterium tuberculosis* H37Rv (locus)	*Kineococcus radiotolerans* SRS30216 (locus)
Ada	O-6-methylguanine/O-4-methylthymine DNA methyltransferase	Ada	No homologs	No homologs	Krad_2866
AlkA	3-methyladenine DNA glycosylase II; DR_2584 is of eukaryotic type	AlkA	DR_2584, DR_2074	Rv1317c	Krad_4325 Krad_3854
AlkB	Alkylation repair protein	AlkB	No homologs	TIGR locus: NT02MT1098	No homologs
Cdc9 (LigB)	ATP-dependent DNA ligase	No homologs	No homologs	Rv3062	Krad_4316
ComEA	DNA uptake protein	ComEA	DR_1855	Rv2415c	Krad_3435
Dam	GATC specific N6-adenine methylase	Dam	No homologs	No homologs	No homologs
Dcd	dCTP deaminase	Dcd	No homologs	Rv0321	Krad_4243
Dcm	Site-specific C-5 cytosine methlytransferase; VSP repair is targeted toward hotspots created by Dcm	Dcm	No homologs	Rv3037c (putative Dcm)	Krad_0734 (putative Dcm)
DinB/DinP	DNA damage inducible protein P (DNA polymerase IV)	DinB	No homologs	Rv3056 Rv1537 (DinX)	Krad_4326 Krad_3213
DinF	Possible DNA-damage-inducible protein F; integral membrane protein; Na^+^-driven multidrug efflux pump	DinF	DR_0792	Rv2836c	Krad_4334
DinG	ATP-dependent DNA helicase; SOS inducer	DinG	No homologs	Rv1329c	Krad_1504
DnaA	Chromosomal replication initiator protein	DnaA	DR_0002	Rv0001c	Krad_0001
DnaB	Replicative DNA helicase	DnaB	DR_0549	Rv0058c	Krad_4333
DnaE	DNA polymerase III (holoenzyme), α subunit	DnaE	DR_0507	Rv1547c Rv3370c	Krad_3187 Krad_3215 Krad_0771 Krad_4598 (on pKRAD01)
DnaG	DNA Primase	DnaG	DR_0601	Rv2343c	Krad_3361
DnaN	DNA polymerase III (holoenzyme), β subunit	DnaN	DR_0001	Rv0002	Krad_1769 Krad_0002
DnaQ	DNA polymerase III (holoenzyme), ε subunit - 3′-5′ exonuclease	DnaQ	DR_0856	Rv3711c	Krad_4419 Krad_3247 Krad_4503 Krad_1768
DnaZ/X	DNA polymerase III (holoenzyme), γ/τ subunit	DnaZ/X	DR_2410	Rv3721c	Krad_0466
Dut	dUTPase	Dut	No homologs	Rv2697c	Krad_1557
ERCC3	XPB/ERCC3 helicase	No homologs	DR_A0131	Rv0861c	Krad_3612
Fmu	rRNA SAM-dependent methyltransferase	Fmu	DR_2168	Rv1407	Krad_2983
Fpg/MutM	Formamidopyrimidine and 8-oxoguanine DNA glycosylase (Homolog of Nei; see below)	Fpg/MutM	DR_0493	Rv2924c Rv0944	Krad_1377 Krad_0158 Krad_0151
FtsK	Chromosome resolution and positioning	FtsK	DR_0400	Rv2748c	Krad_1482
GyrA	DNA gyrase, subunit A	GyrA	DR_1913	Rv0006	Krad_0007
GyrB	DNA gyrase, subunit B	GyrB	DR_0906	Rv0005	Krad_0006
HAM1/YggV	Xantosine triphosphate pyrophosphatase, prevents 6-N-hydroxylaminopurin mutagenesis	HAM1/YggV	DR_0179	Rv1341	Krad_3762
HelD	Helicase IV (ATP-dependent 3′-to-5′ DNA helicase) involved in the RecF pathway of recombination	HelD	DR_1775 (putative UvrD)	No homologs	Krad_0757 (putative UvrD)
HelY	Probable helicase, Ski2 subfamily (ATP-dependent RNA helicase)	No homologs	No homologs	Rv2092c (HelY)	Krad_1885 Krad_0173
HelZ	Probable helicase with a Zinc finger domain, Snf2/Rad54 family	No homologs	DR_1259	Rv2101 (HelZ)	Krad_1013
HsdM	Type I restriction/modification system DNA methylase	HsdM	No homologs	Rv2756c	No homologs
HsdS	Type I restriction/modification system specificity determinant	HsdS	No homologs	Rv2755c (HsdS')	No homologs
HNS	HU-histone protein	HNS	No homologs	Rv3852	No homologs
HolA	DNA polymerase III (holoenzyme), δ subunit	HolA	DR_1244	Rv2413c (unrelated to *E. coli* HolA)	Krad_3422 (unrelated to *E. coli* HolA)
HolB	DNA polymerase III (holoenzyme), δ' subunit	HolB	DR_2332	Rv3644c	Krad_0490
HolC	DNA polymerase III (holoenzyme), chi subunit	HolC	No homologs	No homologs	No homologs
HolD	DNA polymerase III (holoenzyme), psi subunit	HolD	No homologs	No homologs	No homologs
HolE	DNA polymerase III (holoenzyme), theta subunit	HolE	No homologs	No homologs	Krad_2840
HrpA	ATP-dependent helicase	HrpA	DR_0420	No homologs	Krad_3104 Krad_1244
HupB/IHF	DNA binding protein II, Integration host factor (IHF); histone-like proteins	HupB	DR_A0065	Rv2986c(HupB) Rv1388 (IHF)	Krad_2337 Krad_1360 Krad_2805 Krad_2005 Krad_3371
LexA	Transcriptional regulator, repressor of the SOS regulon, autoprotease	LexA	DR_A0344 DR_A0074	Rv2720	Krad_1506
Lhr	ATP-dependent helicase superfamily II	Lhr	No homologs	Rv3296	Krad_1489
LigA	DNA ligase, NAD(+)-dependent	LigA	DR_2069	Rv3014c	Krad_1315
LigB	DNA ligase, NAD(+)-dependent	LigB	No homologs	Rv0938	No homologs
LigC	Probable DNA ligase	No homologs	No homologs	Rv3731 (LigC)	Krad_0653
Mfd	Transcription repair coupling factor; helicase	Mfd	DR_1532	Rv1020	Krad_1067
MPG	3-Methylpurine DNA glycosylase	MPG	DR_2074 (also see AlkA)	Rv1688	Krad_3154
Mrr	Type IV restriction endonuclease	Mrr	DR_1877 DR_0508 DR_0587	Rv2528c	No homologs
Mug (ygjF)	G/T mismatch-specific thymine DNA glycosylase, distantly related to DR_1751; Present as a domain of many multidomain proteins in many eukaryotes	Mug (ygjF)	DR_0715	No homologs	No homologs
MutH	Endonuclease, Component of the MutHLS complex functions in the methyl-directed mismatch repair pathway	MutH	No homologs	No homologs	No homologs
MutS	ATPase, Component of the MutHLS complex functions in the methyl-directed mismatch repair pathway	MutS	DR_1976 DR_1039 contains a frameshift	No homologs	No homologs
MutL	Predicted ATPase, Component of the MutHLS complex functions in the methyl-directed mismatch repair pathway	MutL	DR_1696	No homologs	No homologs
MutT	8-oxo-dGTPase. D.r. encodes additional 17 paralogs; only some predicted to function in repair	MutT	DR_0261	Rv2985c Rv1160c Rv0413c	Krad_1131 Krad_2346 Krad_3140 Krad_2697 Krad_0113
MutY	8-oxoguanine DNA glycosylase & AP-lyase, A-G mismatch DNA glycosylase	MutY	DR_2285	Rv3589c	Krad_0599
Nei	Endonuclease VIII (also see Fpg above)	Nei	No homologs	Rv3297 Rv2464c	Krad_1488 Krad_3521 Krad_0294 Krad_3396
Nfi	Endonuclease V	Nfi	DR_2162	No homologs	No homologs
Nfo	Endonuclease IV (AP endonuclease)	Nfo	No homologs	Rv0670	No homologs
Nth	Endonuclease III & thymine glycol DNA glycosylase; DR_0928 and DR_2438 are of archaeal type and DR_0289 is close to yeast protein	Nth	DR_2438, DR_0289, DR_0928	Rv3674c	Krad_0422
Ogt	O-6-methylguanine/O-4-methylthymine DNA methyltransferase	Ogt	DR_0428	Rv1316c	Krad_3712
ParC	DNA Topoisomerase IV, subunit A (Type II topoisomerase)	ParC	No homologs	No homologs	Krad_1546
ParE	DNA Topoisomerase IV, subunit B (Type II topoisomerase)	ParE	No homologs	No homologs	Krad_1534
PepA	DNA binding (independent of Aminopeptidase activity) protein required for maintenance of plasmid monomers.	PepA	DR_0717	Rv2213	Krad_3276 Krad_1149
PinR	Putative recombinase	PinR	DR_C0005	No homologs	Krad_4707 Krad_4374
PolA	DNA polymerase I	PolA	DR_1707	No homologs	Krad_2951
PolB	DNA polymerase II	PolB	No homologs	No homologs	No homologs
PriA	Putative primosomal protein n' (replication factor Y)	PriA	DR_2606	Rv1402	Krad_2988
PriB	Core component of the primosome, binds to PriA and single-stranded DNA	PriB	No homologs	No homologs	No homologs
PhrB	Photolyase (direct monomerization cyclobutane-type pyrimidine dimers)	PhrB	No homologs	No homologs	Krad_3554 Krad_4047
RadA	Predicted ATP-dependent protease	RadA (Sms)	DR_1105	Rv3585c	Krad_4702
RadC	Predicted acyltransferase; predicted DNA-binding protein	RadC	No homologs	No homologs	No homologs
RarA	Protein may play a role in recombination associated with DNA replication; putative ATPase related to the helicase subunit of the Holliday junction resolvase	RarA	DR_1898	Rv2559c	Krad_3035
RecA	Recombinase; ssDNA-dependent ATPase, activator of LexA autoproteolysis	RecA	DR_2340	Rv2737c	Krad_1492
RecB	Helicase/exonuclease	RecB	No homologs	Rv0630c	Krad_0993
RecB (exo1)	RecB family exonuclease 1	No homologs	No homologs	Rv3202c	Krad_1171
RecB (exo2)	RecB family exonuclease 2	No homologs	No homologs	No homologs	Krad_4407
RecB (exo3)	RecB family exonuclease 3	No homologs	No homologs	Rv2119	Krad_1855
RecC	Helicase/exonuclease	RecC	No homologs	Rv0631c	Krad_0992
RecD	Helicase/exonuclease; Contains three additional N-terminal helix-hairpin-helix DNA-binding modules; closely related to RecD from *B.subtilis* and *Chlamydia*	RecD	DR_1902	Rv0629c	Krad_0994
RecE	Exonuclease VIII	RecE	No homologs	No homologs	No homologs
RecF	Predicted ATPase; required for daughter-strand gap repair	RecF	DR_1089	Rv0003c	Krad_0004
RecG	Holliday junction-specific DNA helicase; branch migration inducer	RecG	DR_1916	Rv2973c	Krad_1368
RecJ	Single-stranded DNA-specific exonuclease	RecJ	DR_1126	No homologs	No homologs
RecN	Predicted ATPase	RecN	DR_1477	Rv1696c	Krad_3147
RecO	Required for daughter-strand gap repair	RecO	DR_0819	Rv2362c	Krad_3375
RecQ	Helicase; suppressor of illegitimate recombination	RecQ	DR_1289 DR_2444	Rv1253	Krad_0829 RecQ-like: Krad_4305 Krad_4391
RecR	Required for daughter-strand gap repair	RecR	DR_0198	Rv3715c	Krad_0465
RecT	DNA annealing protein	RecT	No homologs	No homologs	Krad_1418
RecX	Regulatory protein for RecA	RecX	DR_1310	Rv2736c	Krad_1493
RuvA	Holliday-junction-binding subunit of the RuvABC resolvasome	RuvA	DR_1274	Rv2593c	Krad_3054
RuvB	Helicase subunit of the RuvABC resolvasome	RuvB	DR_0596	Rv2592c	Krad_3053 Krad_3828
RuvC	Endonuclease subunit of the RuvABC resolvasome	RuvC	DR_0440	Rv2594c	No homologs
RusA (YbcP)	Endonuclease/Holliday junction resolvase	RusA (YbcP)	No homologs	No homologs	No homologs
SbcB	Exodeoxyribonuclease I	SbcB	No homologs	No homologs	No homologs
SbcC	Exonuclease subunit, Predicted ATPase	SbcC	DR_1922	No homologs	Krad_2553
SbcD	Exonuclease	SbcD	DR_1921	Rv1277 (unrelated to *E. coli* SbcD)	Krad_0868 Krad_2554 (Both unrelated to *E. coli* SbcD)
SdrA	DNA or RNA helicase of superfamily II	No homologs	No homologs	Rv2917	Krad_3772
Smf	Predicted Rossmann fold nucleotide-binding protein involved in DNA uptake	Smf	DR_0120	Rv2896c	Krad_1409 Krad_4481
SplB	Photolyase	No homologs	No homologs	No homologs	Krad_0827 Krad_4460
SrmB	Superfamily II ATP-dependent helicase	RhlE	DR_0335 DR_1624	Rv3211 Rv1253	Krad_1242 Krad_0858
Ssb	Single-strand binding protein; D. radiodurans R1 has three incomplete ORFs corresponding to different fragments of the SSB	Ssb	DR_0099	Rv0054 Rv2478c	Krad_4338
Tag	3-methyladenine DNA glycosylase I	Tag	No homologs	Rv1210	Krad_0999
TopA/TopB	DNA topoisomerase I (Type IA topoisomerase)/DNA topoisomerase III (Type I topoisomerase)	TopA	DR_1374	Rv3646c	Krad_0487
UDG	Uracil DNA glycosylase	UDG	DR_1751	Rv0322	Krad_3900
UmuC	Error-prone DNA polymerase; in conjunction with umuD and recA, catalyzes translesion DNA synthesis	UmuC	No homologs	No homologs	No homologs
UmuD	In conjunction with UmuC and RecA, facilitates translesion DNA synthesis; autoprotease	UmuD	No homologs	No homologs	No homologs
Ung	Uracil DNA glycosylase; DR_0689 is a likely horizontal transfer from a eukaryote or a eukaryotic virus	Ung	DR_0689 DR_1663	Rv2976c	Krad_3639
Uve1/BS_YwjD	UV-endonuclease; activity was characterized in *Neurospora*	Uve1/BS_YwjD	DR_1819	No homologs	No homologs
UvrA	ATPase, DNA binding	UvrA	DR_1771 DR_A0188	Rv1638c	Krad_2940 Krad_1787 Krad_0057
UvrB	Helicase	UvrB	DR_2275	Rv1633c	Krad_2942
UvrC	Nuclease	UvrC	DR_1354	Rv1420	Krad_2935
UvrD	DNA helicase II (DNA-dependent helicase activity) initiates unwinding from a nick; DR_1572 has a frameshift	UvrD	DR_1775	Rv0949 Rv3198c (putative)	Krad_0757 Krad_1179 Krad_1172 Krad_4408
UvrD2	Putative helicase	UvrD2	DR_1775 (putative UvrD2)	Rv3198c	Krad_1179
Vsr	Strand-specific, site specific, GT mismatch endonuclease; fixes deamination resulting from Dcm	Vsr	No homologs	No homologs	No homologs
XseA/nec7	Exonuclease VII, large subunit	XseA/nec7	DR_0186	Rv1108c	Krad_1122
XseB	Exonuclease VII, small subunit	XseB	DR_2586	Rv1107c	Krad_1121
XthA	Exodeoxyribonuclease III (AP endonuclease)	XthA	DR_0354	Rv0427c	Krad_4198 Krad_3979 Krad_0544
YbaZ	Possible methylated-DNA-[protein]-cysteine S-methyltransferase	YbaZ	DR_0428	Rv3204	Krad_1169
YhdJ	Adenine-specific DNA methylase	YhdJ	DR_C0020	No homologs	No homologs
YejH	DNA or RNA helicase of superfamily II	YejH	DR_A0131_1_2	No homologs	Krad_3612 (see also ERCC3)
YqgF	Predicted Holliday junction resolvase	YqgF	No homologs	Rv2554c	Krad_3031
XerC/XerD	Putative recombinase in segregation of chromosomes, homologous recombination; tyrosine recombinase/inversion of on/off regulator of fimA, FimE/FimB	XerC/XerD	DR_C0018 DR_A0155 DR_B0104	Rv1701 Rv2894c	Krad_3139 Krad_1691
53EXOc	5′-3′ exonuclease; T5 type 5′-3′ exonuclease domains may co-occur with DNA polymerase I (Pol I) domains, or be part of Pol I containing complexes	53EXOc	DR_1707	Rv2090	Krad_1887
	Predicted eukaryotic-type DNA primase	No homologs	No homologs	Rv3730c Rv0269c	Krad_0652 Krad_4154
	Predicted site-specific integrase-resolvase	No homologs	No homologs	Rv3750c Rv2310	Krad_0047

Genes involved in carotenoid biogenesis have been shown to confer a modest level of radiation resistance [13]–[15] by scavenging electrons from reactive oxygen species (table 1). *K. radiotolerans* produces carotenoids [2] and the carotenoid biosynthetic pathway is similar to that found in other organisms. In addition to the two genes listed in table I encoding phytoene synthetase and phytoene desaturase, *K. radiotolerans* produces polyprenyl synthase (Krad3227), lycopene cyclase (*crtY*, Krad0091), and neurosporene dehydrogenase (*crtD*, Krad3225). A hydroxlase gene (*crtZ*) was not discovered suggesting that the *K. radiotolerans* carotenoids are not hydroxylated.

Higher eukaryotes repair DSB using nonhomologous end joining (NHEJ) ([16], [17]Riha et al. 2006). NHEJ repair is mediated by the Ku complex and the Ligase IV/XRCC4 complex along with other proteins whose precise biochemical functions remain to be elucidated. While *E. coli* lacks the NHEJ pathway, some Actinobacteria genera such as *Mycobacterium* have this repair pathway. Mycobacterial Ku binds DNA ends and recruits a polyfunctional DNA ligase/polymerase (LigD) *in vitro* (Della et al. 2004). Though repair is mutagenic, it does help maintain cell viability and loss of Ku and LigD increases sensitivity to ionizing radiation (Stephanou et al. 2007). *K. radiotolerans* appears to lack a Ku-like DNA binding protein. While it does encode an ATP-dependent DNA ligase (Cdc9, or LigB; table 2) NHEJ repair is not likely without Ku.

Ionizing radiation also causes many other types of DNA damage. The *K. radiotolerans* DNA replication, recombination and repair gene set is overlapping with [9], but generally different from those of *D. radiodurans* [18] and *E. coli* [19] which may be expected since these organisms belong to different phyla (table 2). As one illustration of this difference, *K. radiotolerans* but not *D. radiodurans* contains RecB and RecC which are involved in recombinational repair in *E. coli* (reviewed in [20]). DNA replication, repair and recombination systems in *K. radiotolerans* are more similar to those of *Mycobacterium tuberculosis* [21], possibly a reflection of the phylogenetic proximity of the two organisms. In addition, *M. tuberculosis* is at least 10-fod more resistant to ionizing radiation than *E. coli* [22]. Because *M. tuberculosis* is an actively studied pathogen its genome is well-annotated. Because it has coevolved the majority of its DNA replication, repair, and recombination mechanisms with *K. radiotolerans* we chose it as a reference organism, together with *E. coli* and .

Both *K. radiotolerans* and *M. tuberculosis* lack the classical bacterial mismatch repair genes MutS, MutH, MutL, RecJ, ExoVIII, ExoI and Mug (table 2). A compensating factor may be production of DNA polymerases with increased fidelity and proofreading efficiency as *K. radiotolerans* contains four exonuclease (ε; DnaQ) subunits, three polymerase (α; DnaE) subunits and two β (DnaN) subunits of the replicative DNA polymerase III. Mismatch repair in *K. radiotolerans* may also be handled by proteins unrelated to classical bacterial mismatch repair proteins. Some base excision repair genes that may take on such roles are also uniquely overrepresented. There are three Fpg and four Nei base excisionases in *K. radiotolerans*, compared with one Fpg and no Nei homologs in *D. radiodurans*. As an additional example of divergence with *D. radiodurans*, 3-methyladenine DNA glycosylase I (Tag) is present in *K. radiotolerans*, but absent from *D. radiodurans*.

The nucleotide excision repair pathway is also overrepresented in *K. radiotolerans* by three *uvrA* orthologs and by five genes encoding UvrD-like helicases. In addition to these helicases, the *K. radiotolerans* genome, similarly to *M. tuberculosis*, contains an ERCC3 (XPB)-like superfamily II helicase, whose eukaryotic homolog performs essential functions in nucleotide excision repair and transcription [23]. The *K. radiotolerans* and *M. tuberculosis* XPB helicases have been recently demonstrated to be functional *in vitro* (Biswas and Tsodikov, unpublished).

Five homologs of histone-like proteins (IHF or HupB-like) may package DNA in order to protect it from damage, aid recombinational repair, or maintain multiple chromosomes. The direct reversal pathway used to repair pyrimidine dimers includes two copies of the *phrB* photolyase gene and an additional, *splB* photolyase gene, which is absent in *E. coli*, *D. radiodurans* and *M. tuberculosis.* Other differences from *M. tuberculosis* include the presence in *K. radiotolerans* of PolA, Topo IV, HolIII, YejH, which are absent in *M. tuberculosis*. The translesion DNA repair system UmuC/UmuD, present in *E. coli* but not *D. radiodurans* or *M. tuberculosis*, is also a part of the *K. radiotolerans* genome.

Similar to mycobacteria, the *K. radiotolerans* replication gene set lacks a well-defined homolog of the helicase loader (DnaC), but contains the other main replication genes (DnaA, DnaB, DnaG). *K. radiotolerans* and *M. tuberculosis* both contain a eukaryotic-like DNA primase gene.

In summary, many of the genes known to confer radiation resistance in *D. radiodurans* are missing from *K. radiotolerans* suggesting novel components to the repair and protection toolbox. Two pathways are involved in DSB repair in other organisms, ESDSA repair mediated by RecA and PolA and NHEJ repair mediated by Ku and LigD. RecA and PolA are present and may be aided by the presence of RecB and RecC. *K. radiotolerans* lacks the Ku protein making the presence of the NHEJ pathway unlikely. Base excision and nucleotide excision repair pathway genes are over represented in *K. radiotolerans* relative to other bacteria.

### Reactive oxygen species detoxification

Most organisms have both RecA and PolA without exhibiting extreme radiation resistance. Remarkably, *D. radiodurans cells* rendered ionizing radiation-sensitive by a *polA* mutation are fully complemented by expression of the *polA* gene from ionizing radiation-sensitive *E. coli* [24]. Repair proteins, either native or cloned, may function better after irradiation in *D. radiodurans* cells due to protection from protein oxidation [25]. The genetic components and molecular mechanisms of protein repair/protection remain unknown but appear to be correlated with a Mn/Fe ratio in the range of 0.12–0.37 [26]. The Mn/Fe ratio in *K. radiotolerans* is 0.09, slightly lower than *Deinococcus* but much higher than that of radiation sensitive organisms [27].

*D. radiodurans* is adept at detoxifying reactive oxygen species (ROS) during radiation exposure when many free radicals are generated from hydrolysis of water [13]–[15]. Like *Deinococcus*, *K. radiotolerans* produces carotenoids as one level of protection. *K. radiotolerans* also possesses an impressive suite of genes involved in ROS detoxification (table 3) comparable to those found in bacterial pathogens of mammals such as *Neisseria gonorrhoeae* [28]. Pathogenic bacteria are routinely exposed to oxidative stress by the host as part of an innate immune response. These results suggest a thorough ROS detoxification network.

**Table 3:** Reactive oxygen species detoxification genes in *K. radiotolerans.*

Enzyme	Krad Locus Tag
Alkyl hydroperoxide reductase, *ahpC*	3757
Catalase, *katE*	0865
Mn Catalase, *katA*	0815
Cytochrome c peroxidase, *mauG*	Absent
Glutathionyl spermidine synthase, GSP-Syn	2757
Glutathione peroxidase, GSHPx	1247
Dyp-type peroxidase	3350
Organic hydroperoxidase	0813
Peptide methionine sulfoxide reductase, *msrA*	1091
Fe/Mn superoxide dismutase, *sodA*	3578**
Superoxide reductase, *sorA*	Absent

### Bioremediation potential

Because *K. radiotolerans* was isolated from a HLW facility, the genome was examined for gene products that could be used to generate carbon or energy from organic compounds in the tanks. Orthologs belonging to known degradation pathways were not detected for U.S. Departments of Energy (DOE) and Defense (DOD) selected priority pollutants for including benzene, toluene, ethylbenzene, and xylenes (the BTEX compounds), chlorinated hydrocarbons, polynuclear aromatic hydrocarbons, polychlorinated biphenyls, ketones, alkanes, phenols, phthalates, explosives, and pesticides. Several homologues for atrazine degradation were noted (*AtzABZ,* Krad1947, 4281, 0533, respectively), though the pathway seems incomplete.

The SRS HLW also contains low molecular weight organic complexants which interfere with downstream chemical stabilization and processing. Commonly used complexants and decontamination reagents at the SRS include oxalate, glycolate, citrate, and formate. Growth using each organic acid as a sole carbon source was evaluated for *K. radiotolerans* in comparison with glucose as a positive control and 0.1% yeast extract as a negative control. Respiration was examined using O_2_ consumption and CO_2_ evolution. Preliminary studies revealed that citrate and glycolate were unsuitable growth substrates (data not shown). Respiration rates and biomass yields on glucose were higher than those of other carbon sources (table 4). Biomass yields with 5 mM oxalate and 5 mM formate were generally consistent with the 0.1% yeast extract control though higher concentrations were toxic. Similarly, O_2_ consumption and CO_2_ production were both better at lower oxalate and formate concentrations. While biomass production was similar between formate, oxalate, and YE, the respiration rates were higher for formate and oxalate.

**Table 4:** Metabolism of glucose, oxalate and formate by *K. radiotolerans*.

Substrate	Consumption/Production Rates (µmol/hr)			Dry Weight (mg)
	O2	CO2	O2:CO2	
Glucose (5mM)	8.08	6.71	1.2	2.77±0.21
Glucose (10mM)	8.72	7.87	1.11	3.05±0.07
Oxalate (5mM)	1.83	1.36	1.35	2.17±0.06
Oxalate (10mM)	1.42	1.09	1.31	1.73±0.21
Formate (5mM)	6.43	6.36	1.01	2.30±0.10
Formate (10mM)	5.87	5.75	1.02	1.43±0.25
YE (0.1%)	4.97	4.53	1.1	2.20±0.00
Kill Control	0	0	0	0.00±0.00

**Figure 5:**
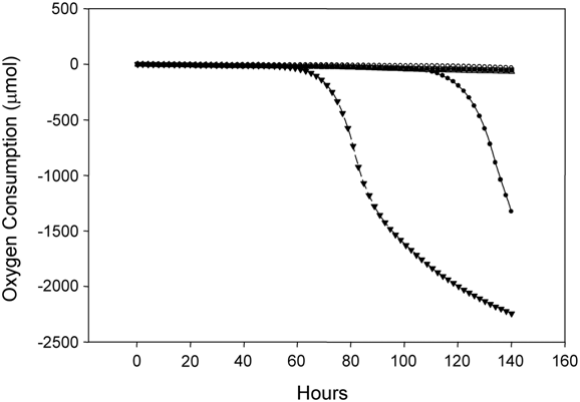
Substrate-facilitated survival and recovery of *K. radiotolerans* following starvation. Following 7 days of incubation, cultures starved in the presence of oxalate (▾) and yeast extract (•) recovered following transfer to fresh TGY medium, while no respiratory response was measured from cultures starved in the presence of glucose, formate, citrate, or Tris buffer.

While none of the carbon sources stimulated growth, formate and oxalate promote survival and quicken recovery of *K. radiotolerans* following prolonged starvation (table 5). In these experiments, chloramphenicol was used to accelerate starvation and to uncouple growth from survival. At intervals of 3, 7 or 14 days cells were resuspended in fresh TGY medium and respiration measured. After three days of incubation, substrate specific differences were already apparent. Once starvation was lifted, the highest respiration rates were recorded for cells starved in the presence of oxalate and formate. While all of the cultures recovered, the formate-starved culture was first to enter exponential growth, 4 hours ahead of all other treatments (data not shown). Remarkably, after 7 days of starvation, the highest rate of respiration was measured from the oxalate treated culture, which recovered and began exponential growth 60 hours after starvation was relieved (Figure 5). The yeast extract treated culture also recovered following more than 100 hours of post-starvation recovery. None of the cultures responded after 14 days of treatment.

**Table 5:** Starvation recovery of *K. radiotolerans*.

	Starvation Recovery (respiration rates, µmol/hr)					
	3 Days		7 Days		14 Days	
Substrate	O2	CO2	O2	CO2	O2	CO2
Glucose	8.63	50.42	0.25	0.15	0.3	0.09
Formate	19.57	125.9	0.49	0.22	0.3	0.05
Oxalate	11.02	58.09	15.99	14.21	0.3	0.08
Citrate	2.65	11.32	0.37	0.13	0.3	0.05
YE (0.01%)	1.29	1.12	9.47	8.54	0.07	0.09

*E. coli* contains three formate dehydrogenases. Formate dehydrogenase H (FdnF is coupled to a hydrogenase and used for hydrogen production by the formate hydrogen lyase reaction. *K. radiotolerans* appears to have this enzyme (Krad1331). Formate dehydrogenase N (FdnGHI) is induced when cells are grown anaerobically with nitrate. Formate dehydrogenase O (FdoGHI) is expressed aerobically and may participate in transfer of electrons from formate to oxygen. Formate dehydrogenases N and O have similar amino acid sequences. *K. radiotolerans* has one of the two (Krad1601, Krad1602, Krad1603), but it is difficult to tell based on the amino acid sequence whether it is an N-type or an O-type.

The *K. radiotolerans* genome encodes glycolysis, pentose phosphate, tricarboxylic acid (TCA) cycle and glyoxylate bypass pathways (table 6). The pentose phosphate cycle [29] and the glyoxylate bypass [30] may both be involved in radiation resistance and post-irradiation recovery as in *D. radiodurans*. Both pathways are important routes for the production of biosynthetic metabolites while minimizing the production of damaging oxygen radicals. *K. radiotolerans* exhibits aerobic respiration on 5- and 6-carbon sugars (eg., fructose, amylose, maltose) and sugar alcohols (eg., glycerol, mannitol, sorbitol) (data not shown). A partial gluconeogenesis pathway was identified (missing pyruvate carboxylase, *pcb*, and glucose-6-phosphatase, *glp*). A partial Entner-Doudoroff pathway was also found, lacking glucose dehydrogenase, *gdh*, and phosphogluconate dehydratase, *edd*. Carbohydrate metabolism is supported by many ABC-type sugar transporters.

**Table 6:** Central metabolism in *K. radiotolerans.*

Pathway	Krad Locus Tag	Gene Designation	Gene Product Function
Glycolysis	3239	*glcA*	Hexokinase
	1463	*gpi*	Glucose-6-phosphate isomerase
	4274	*pfkA*	Fructose-6-phosphate kinase
	2157	*fba*	Fructose-1,6-bisphosphate aldolase
	2931	*gapA*	Glyceraldehyde-3 phosphate dehydrogenase
	2930	*pgk*	Phosphoglycerate kinase
	0891	*pgm*	Phosphoglyceromutase
	1073	*eno*	Enolase
	2959	*pyk*	Pyruvate kinase
Gluconeogenesis	3372	*ppc*	Phosphoenolpyruvate carboyxkinase
	1463	*pgi*	Phosphoglucoisomerase
	Absent	*pcb*	Pyruvate carboxylase
	Absent	*glp*	Glucose-6-phosphatase
TCA Cycle	1140	*gltA*	Citrate synthase
	1566	*acnA*	Aconitase
	3988	*icd*	Isocitrate dehydrogenase
	1228	*sucA*	Oxoglutarate dehydrogenase
	3279	*sucB*	Succinyl-transferase
	3999	*sucC*	Succinyl-CoA synthetase, beta
	3998	*sucD*	Succinyl-CoA synthetase, alpha
	3955	*frdB*	Fumarate reductase
	3954	*sdhA*	Succinate dehydrogenase
	1112	*fumC*	Fumarase
	0742	*mdh*	Malate dehydrogenase
Entner-Doudoroff	1494	*gnl*	Gluconolactonase
	0597	*gntK*	Gluconate kinase
	2133	*eda*	2-keto-3-deoxy-6-phosphogluconate aldolase
	Absent	*edd*	Phosphogluconate dehydratase
	Absent	*glp*	Glucose dehydrogenase
Pentose Phosphate Cycle	0133	*zwf*	Glucose-6-phosphate dehydrogenase
	2926	*pgl*	6-Phosphogluconolactonase
	0003	*gnd*	6-Phosphogluconate dehydrogenase
	2922	*tktA*	Transketolase
	2923	*tal*	Transaldolase
	1918	*rpiB*	Ribose 5-phosphate isomerase
	2980	*rpe*	Ribulose phosphate 3-epimerase
Glyoxalate Bypass	0108	*aceA*	Isocitrate lyase
	2227	*aceB*	Malate synthase

The *K. radiotolerans* genome was queried by the tblastn algorithm using reference sequences from the genomes of *Frankia alni* ACN14a, *Streptomyces coelicolor* A3(2), *Deinococcus radiodurans* R1, and *E. coli* K12. Cut-off values for confident identification required both amino acid percent identities >45% and an E-score < e-10.

Electron acceptors other than O_2_ increase habitat diversity by enabling growth when oxygen tension is low. Krad1328 has strong identity with *Arthrobacter aurescens* TC1 nitrate reductase. Nitrate respiration may be a general feature of *Arthrobacter* species as *Arthrobacter globiformis* and *Arthrobacter nicotianae* have been shown to utilize NO_2_^−^ as a terminal electron acceptor [31]. *Arthrobacter* spp. are routinely recovered from subsurface environments contaminated by high level radioactive waste [32], [33]. Krad1328 is not related to the *Kuenenia stuttgartiensis* enzyme that participates in anammox, anaerobic ammonium oxidation during which nitrite and ammonium are converted directly into dinitrogen gas.

The *K. radiotolerans* genome possesses a large number of molybdopterin oxidoreductases. Krad4344 has striking identity to the trimethylamine-N-oxide (TMAO) reductase TorA from *E.coli* K12, which is a terminal electron acceptor that is specific for N-oxides. TorA is connected to the quinone pool via a membrane-bound penta-haem cytochrome (TorC) [34]. DorCA in *Rhodobacter* is very similar to TorCA with the difference that DorA catalyses reduction of both dimethylsulfoxide and TMAO. *K. radiotolerans* lacks a homolog to TorC or DorC and reduction of TMAO has not been experimentally examined. There is no evidence for the type of fumarate reductase that reduces fumarate to succinate during anaerobic growth or for proteins homologous those from *Shewanella* and *Geobacter* involved in metal reduction.

In summary, *K. radiotolerans* exhibits moderate nutritional versatility with genes for growth on a variety of carbon sources and at least two terminal electron acceptors. This organism appears to utilize two of the commonly used complexants, oxalate and formate. These results suggest that *K. radiotolerans* should be investigated for use in scrubbing complexants from HLW tanks. The ability of formate and oxalate to stimulate respiration and prolong viability might provide certain clues into the metabolic ecology of *K. radiotolerans*. *Kineococcus* species are commonly found on weathered rock, stone monuments, paintings, desert crust or varnish along with cyanobacteria and fungi which together form biotic crusts, lichens, or endolithic communities (Garrity et al 1996, Gómez-Silva et al 2008, Imperi et al 2007, Kuhlman et al 2006, Schabereiter-Gurtner et al 2001, Torre et al 2003, Tringe et al 2005). Cyanobacteria, fungi, as well as plants, release formate and oxalate for toxic metal chelation and detoxification, and mineral dissolution (Neaman et al., 2005;West and Coombs, 1981). Several studies have demonstrated heterotrophic mineralization of oxalate to CO_2_ without assimilation but the mechanism remains unknown.

### Life cycle

The production of motile zoospores is widespread but patchy among members of the Actinobacteria phylum. All three *Kineococcus* species produce zoospores during part of their life cycle, but zoospore formation has not been extensively examined in any species [2], [35], [36]. Zoospores are produced at the tips of substrate hyphae and in clusters on sporophores in the closely related genus *Kineosporia* [37]. However, *K. radiotolerans* cells do not produce hyphae or sporangioles. Cells grown in rich PYTG medium produce large clusters of nonmotile cells that alternate division planes to form cubes of cells connected by a thick extracellular matrix [2]. During stationary phase spherical, dispersed zoospores, approximately 1 µm in diameter, are produced. In PTYG medium, the spores lack flagella. Motile spores were observed only after exposure to 10% sandy loam extract or with certain carbon sources [2]. Thus the production of zoospores and the development of flagella on those spores are subject to environmental cues that remain to be elucidated.

Movement of *Kineosporia* SR11 zoospores up chemical gradients has been observed for a variety of inorganic compounds [38]. Remarkable speeds of up to 160 µm s^−1^ are achieved in *Kineosporia* SR11 zoospores [38]. *Kineospora* SR11 is attracted to K^+^, Mg^+2^, and Ca^+2^ salts of phosphate, sulfate and halides, but not other combinations of these ions, suggesting that chemotaxis may be dependent on specific cations and anions pairs. The *K. radiotolerans* genome contains complete pathways for flagellar biogenesis and chemotaxis suggesting that zoospore dispersal is designed to colonize new niches for growth.

The bacterial flagellum is a complex nanomachine that requires dozens of gene products for its assembly and function. Flagellar gene expression is temporally regulated to produce proteins as they are needed for flagellar assembly. Where it has been examined, temporal control of flagellar gene expression is achieved through a transcriptional hierarchy initiated by a master regulator [39], [40]. Flagellar genes in *K. radiotolerans* are clustered within a motility island located between Krad1621 and Krad1673 (table 7). Genes within this region encode components of the basal body, hook, filament, flagellar protein export apparatus, flagellar chaperones, flagellar motor and motor switch. None of the genes within the motility island encode proteins that share homology with known master regulators of flagellar biogenesis though some of the genes encode proteins involved in regulating expression of flagellar biogenesis or motility. One such regulatory protein is a FliA (σ^28^) homolog encoded by Krad1625. FliA is an alternative σ factor that is required for transcription of flagellar genes in many bacteria whose products are needed late in flagellar assembly [39], [40]. Activity of FliA is often regulated by anti-sigma factor FlgM [39]–[41]. Upon completion of the hook-basal body complex FlgM is secreted from the cytoplasm via the flagellar protein export apparatus, which allows expression of the FliA-dependent flagellar genes. Bacteria that possess a *fliA* ortholog often also possess an ortholog of *flgM* [42], suggesting that negative control of FliA activity by FlgM is a general feature of flagellar gene regulation. *K. radiotolerans* does not possess a *flgM* ortholog, nor do the other flagellated Actinobacteria sequenced to date (*Norcardioides* sp. JS614, *Acidothermus cellulolyticus* and *Leifsonia xyli*). It is possible that *K. radiotolerans* possesses a protein that is functionally equivalent to FlgM but does not share homology with it. Alternatively, regulation of FliA may occur through a novel mechanism in *K. radiotolerans* and related Actinobacteria.

**Table 7:** Flagellar genes from *K. radiotolerans*.

Krad Locus tag	Gene designation	Gene product function
1606		Methyl-accepting chemotaxis protein
1613		Methyl-accepting chemotaxis protein
1621	*flgL*	Hook-associated protein 3
1622	*flgK*	Hook-associated protein
1625	*fliA*	Sigma-28
1626	*fliC*	Flagellin
1627	*fliD*	Flagellar capping protein
1628	*fliS*	Flagellin-specific chaperone
1647	*flgB*	Rod protein
1648	*flgC*	Rod protein
1649	*fliE*	MS-ring/rod linker
1650	*fliF*	MS-ring protein
1651	*fliG*	Motor switch protein
1652	*fliH*	Export apparatus component
1653	*fliI*	ATPase for export apparatus
1656	*fliK*	Hook-length control protein
1657	*flgD*	Hook capping protein
1658	*flgE*	Hook protein
1659	*flbD* family member	Function unknown
1660	*motA*	Flagellar motor component
1661	*motB*	Flagellar motor component
1662	*fliL*	Basal body protein, function unknown
1663	*fliM*	Motor switch protein
1664	*fliN*	Motor switch protein
1665	*fliO*	Export apparatus protein
1666	*fliP*	Export apparatus protein
1667	*fliQ*	Export apparatus protein
1668	*fliR*	Export apparatus protein
1669	*flhB*	Export apparatus protein
1670	Possible *cckA* homolog	Histidine kinase; GAF domain protein
1671		Histidine kinase; GAF domain protein
1672		Receiver domain protein
1673	*flhA*	Export apparatus component
1677	*csrA*	Carbon storage regulator

A second regulatory gene within the *K. radiotolerans* motility island is *csrA*, which encodes the carbon storage regulator. The *csrA* gene is also associated with flagellum biosynthetic genes in *Norcardioides* sp. JS614, *A. cellulolyticus* and *L. xyli*. CsrA is a global regulator that binds mRNA to regulate gene expression either positively or negatively [43], [44]. CsrA-mediated control of flagellar biogenesis and/or motility has been demonstrated in some Gram-negative bacteria [45]–[47]. CsrA stimulates expression of *E. coli flhDC* (encodes the master regulator for flagellar biosynthesis) by binding the *flhDC* operon leader transcript and stabilizing the mRNA [47]. CsrA was recently shown to repress expression of the *Bacillus subtilis* flagellin gene, *hag*, by preventing ribosome binding to the transcript [48]. RNA footprinting assays identified two CsrA-binding sites in the *hag* transcript, one of which (BS2) overlaps the Shine-Dalgarno sequence. The corresponding region of the predicted transcript of the *K. radiotolerans* flagellin gene (Krad1626) matches well to the BS2 site of *B. subtilis hag* (9 of 12 bases match). Thus, CsrA may regulate expression of the *K. radiotolerans* flagellin gene as it does *B. subtilis hag*.

Developmental control of flagellar genes has been extensively examined during the dimorphic life cycle of *Caulobacter crescentus*, where a non-flagellated, stalked mother cell undergoes asymmetric binary fission to produce a flagellated swarmer cell [49]–[51]. The swarmer cell eventually differentiates into a mother cell, losing its flagellum and elaborating a stalk. Several genes involved in developmental regulation of flagellar biogenesis in *C. crescentus* have been identified [49]–[51] including *cckA*, a histidine kinase in an operon of export genes that activates the master regulator, CtrA [52]. Krad1670, a predicted histidine kinase, appears to be the last gene of an operon that includes *fliA*, *fliR* and *flhB*, genes encoding components of the flagellar protein export apparatus. Given this conservation in synteny *K. radiotolerans* Krad1670 may play a role in developmental regulation of flagellar biogenesis similar to that of *cckA* in *C. crescentus*.

Signal transduction in *K. radiotolerans* has been examined using the microbial signal transduction database MiST [53]. *K. radiotolerans* exhibits regulatory complexity comparable to that of *Streptomyces spp.* with regard to two-component systems. *K. radiotolerans* is unusual in having 116 di-guanylate cyclase domains. In general these protein domains, which are used to synthesize the second messenger cyclic di-GMP, are over represented in members of the Frankineae suborder of the Actinobacteria but even still *K. radiotolerans* exhibits large over representation. These results suggests extensive transcriptional regulation by cyclic di-GMP, possibly through the use of riboswitches [54].

### Conclusions

Exposure to extreme levels of ionizing radiation does not occur naturally on this planet. While natural nuclear reactors may have existed on Earth about 2 billion years ago, for example in the Oklo region of Gabon, the extreme radiation resistance observed today is more likely to be an evolutionary response to desiccation, which challenges cell viability in a manner similar to ionizing radiation [55]. Like many other radiation resistant bacteria *K. radiotolerans* exhibits resistance to desiccation [2]. Nevertheless, such organisms may have tremendous potential in remediating metabolizable organic constituents of HLW. Removal of organic constituents directly in HLW tanks could greatly improve processing efficiency of HLW. *K. radiotolerans* appears to utilize two of the commonly used complexants at the SRS, oxalate and formate, and should be investigated for use in scrubbing complexants from HLW tanks.

## Materials and Methods

### Culture conditions and chemicals

*Kineococcus radiotolerans* SRS30216 (BAA-149) was obtained from the American Type Culture Collection (ATCC; Manassas, VA, USA). Cultures were maintained on TGY plating medium (1.0% tryptone, 0.1% glucose, 0.5% yeast extract, 1.4% Difco agar). Except where noted all experiments were conducted in semi-defined growth medium (6.1 g/L Tris (pH 7.0), 0.1% YE) amended with different carbon sources to a final concentration of 5 mM unless otherwise mentioned. All cultures were grown at 28°C and liquid cultures were shaken at 150 rpm.

### Genome sequencing

The genome of *Kineococcus radiotolerans* SRS30216 was sequenced at the Joint Genome Institute (JGI) using a combination of 3 kb, 8 kb and 340 kb DNA libraries. All general aspects of library construction and sequencing performed at the JGI can be found at <http://www.jgi.doe.gov/>. Draft assemblies were based on 70,886 total reads. All three libraries provided 9.5× coverage of the genome. The Phred/Phrap/Consed software package (<http://www.phrap.com>) was used for sequence assembly and quality assessment (Ewing and Green 1998; Ewing et al. 1998; Gordon et al. 1998).

After the shotgun stage, 23,746 reads were assembled with parallel phrap (High Performance Software, LLC). Possible misassemblies were corrected with Dupfinisher (Han, 2006,) clone shatter libraries or transposon bombing of bridging clones (Epicentre Biotechnologies, Madison, WI). Gaps between contigs were closed by editing in Consed, custom primer walks, or PCR amplification (Roche Applied Science, Indianapolis, IN ). A total of 4392 primer walk reactions, 5 transposon bombs, 28 clone shatter libraries, and 4 PCR shatter libraries were necessary to close gaps, to resolve repetitive regions, and to raise the quality of the finished sequence. The completed genome sequences of *K. radiotolerans* SRS30216 contains 71,381 reads, achieving an average of 14-fold sequence coverage per base with an error rate less than 1 in 100,000. The *K. radiotolerans* SRS30216 genome has 3 contigs. The main chromosome and the largest plasmid, pKrad1, both appear to be linear. PCR and blunt end adapter PCR were used unsuccessfully to attempt to close the gaps.

### Southern blotting and PCR

A culture of *Kineococcus radiotolerans* was grown to mid log phase in 100 ml of PTYG (0.5% (w/v) yeast extract, 0.5% (w/v) tryptone, 0.5% (w/v) peptone, 0.006% (w/v) MgSO_4_, 0.0006% (w/v) CaCl_2_). The cells were collected by centrifugation at 10,000 × g for five minutes, resuspended in 9.5 ml TE buffer (10 mM Tris-HCl, 1 mM EDTA, pH 8.0), and frozen at −20°C for approximately one hour. The cells were quickly thawed at 37°C, mixed with 0.5 ml of 10% SDS and 50 µl of 20 mg/ml proteinase K, and incubated at 37°C for one hour. Then 1.8 ml of 5 M NaCl was added along with 1.8 ml of 1 g CTAB (Hexadecyltrimethylammonium Bromide) and 10 ml of 0.7 M NaCl. The mixture was incubated for 20 minutes at 65°C, extracted with an equal volume of chloroform/isoamyl alcohol (24∶1), and centrifuged for 10 minutes at 10,000 × g at room temperature. The aqueous upper phase was transferred to a fresh tube and the DNA precipitated with 0.6 volume of isopropanol then washed with 70% ethanol. The pellet was resuspended in 2 ml TE buffer and incubated at 37°C with 40 µg/ml DNase free RNase for at least 1 hr.

The sample was extracted with an equal volume of Tris buffered phenol (pH 7.9) and the upper aqueous phase was transferred to a fresh tube, where it was extracted with an equal volume of 1∶1 phenol and chloroform and then an equal volume of 24∶1 chloroform and isoamyl alcohol. The DNA was precipitated with 0.5 M ammonium acetate and 60% ethanol at −20°C for 20 minutes and then pelleted. The pellet was washed with 70% ethanol and allowed to air dry. The pellet was resuspended in 0.5 ml TE buffer and the DNA concentration was about 1 µg/ml.

Probes were created for each theoretical end of the *K. radiotolerans* chromosome using PCR and digoxygenin tnucleotides (Roche Biochemicals). Primers Krad2223F 5′CGGCAGATACCGGGTTTGATGTTC3′ and Krad2223R 5′CGATGTCGGTGCGGCTGTAGC3′ generated a DNA fragment that was homologous with Krad2223. The PCR reaction contained 0.1 ng of genomic DNA, 0.5 µM of each primer solution. The genomic DNA was initially denatured 2 minutes at 95°C. In subsequent cycles the DNA was denatured for 30 seconds at 95°C, the primers were annealed at 60°C for 30 seconds, and the DNA extended at 72°C for 90 seconds. After 30 cycles the final extension was at 72°C for 3 minutes**.** The PCR products were separated by agarose gel electrophoresis to determine the success of the PCR reaction.

Genomic *K. radiotolerans* DNA was digested with restriction enzymes, BamHI, BglII or NcoI as specified by the manufacturer. The restriction fragments were separated by electrophoresis in 0.8% agarose. The gel was submerged in denaturing solution (0.5 M NaOH, 1.5 M NaCl) for 30 minutes and neutralized (0.5 M Tris-HCl, 1.5 M NaCl, pH 7.5) for 30 minutes. The contents of the gel were transferred to a nylon membrane (Roche Biochemicals) using 20× SSC (0.3 M sodium citrate, 3 M NaCl, pH 7.0). The membrane was exposed in a UV crosslinker. Hybridizations were performed in roller bottles with 20 ml hybridization solution and 15 µl probe at 60°C overnight as recommended by the manufacturer (Roche Biochemicals). CPSD was added to the membrane, incubated at 25°C for 10 minutes and 37°C for 15 minutes, then exposed to X-ray film for 1–10 minutes.

### Starvation Experiments

*K. radiotolerans* was grown in TGY medium to mid-exponential phase (∼26 hr) at 28°C and 150 rpm. Biomass was harvested by centrifugation, washed in 1× PBS (137 mM NaCl, 2.7 mM KCl, 10 mM Na_2_HPO_4_, 2 mM KH_2_PO_4_), and split evenly into different tubes containing 50 mM Tris HCl (pH 7.0), 0.01% yeast extract, chloramphenicol (100 µg/ml), and an individual substrate at a final concentration of 5 mM. Cultures were incubated at 28°C. After 3, 7, and 14 days of imposed starvation, the biomass was harvested, suspended in fresh TGY medium (50 ml), and the ability of each culture to recover and resume growth was compared across substrates.

### Respirometry

Metabolic rates were measured using the Micro-Oxymax multichannel respirometer (Columbus Instruments; Columbus, OH, USA). Respirometry bottles (250 ml capacity) were filled with 50 ml of semi-defined growth medium and headspace gases were analyzed every 2 hr. Bottles were incubated at 28°C and 100 rpm. The Micro-Oxymax respirometer measures the volume of gas consumed (O_2_) or produced (CO_2_). Conversions were made to moles of gas using the ideal gas law. End point biomass determinations were made in 125 ml shake flasks using 25 ml of growth medium. Cells were filtered onto 0.22 µm GS filters (Millipore) and dried to a constant weight at 65°C.
